# Recommendations for pre-symptomatic genetic testing for hereditary transthyretin amyloidosis in the era of effective therapy: a multicenter Italian consensus

**DOI:** 10.1186/s13023-020-01633-z

**Published:** 2020-12-14

**Authors:** M. Grandis, L. Obici, M. Luigetti, C. Briani, F. Benedicenti, G. Bisogni, M. Canepa, F. Cappelli, C. Danesino, G. M. Fabrizi, S. Fenu, G. Ferrandes, C. Gemelli, F. Manganelli, A. Mazzeo, L. Melchiorri, F. Perfetto, L. G. Pradotto, P. Rimessi, G. Tini, S. Tozza, L. Trevisan, D. Pareyson, P. Mandich

**Affiliations:** 1 Department of Neuroscience, Rehabilitation, Ophthalmology, Genetics and Maternal and Child Health (DINOGMI), Section of Medical Genetics, University of Genoa, c/o DIMI Viale Benedetto XV, 6, 16132 Genova, Italy; 2 IRCCS Policlinico San Martino, Genova, Italy; 3 Amyloidosis Research and Treatment Center, IRCCS Fondazione Policlinico San Matteo, Pavia, Italy; 4 UOC Neurologia, Fondazione Policlinico Universitario A. Gemelli IRCCS, Rome, Italy; 5 Università Cattolica del Sacro Cuore, Rome, Italy; 6 Department of Neuroscience, University of Padova, Padova, Italy; 7 Medical Genetics, Azienda Sanitaria Dell’Alto Adige, Bolzano, Italy; 8 Centro Clinico Nemo Adulti-Fondazione Policlinico Universitario Agostino Gemelli IRCCS, Rome, Italy; 9 Cardiovascular Disease Unit, IRCCS Policlinico San Martino, Genova, and IRCCS Italian Cardiovascular Network, Department of Internal Medicine, University of Genova, Genova, Italy; 10 Tuscan Regional Amyloidosis Center, Careggi University Hospital, Firenze, Italy; 11 Molecular Medicine Department, University of Pavia, Pavia, Italy; 12 Department of Neurosciences, Biomedicine and Movement Sciences, Section of Neurology, University of Verona and University Hospital GB Rossi, Verona, Italy; 13 Unit of Rare Neurodegenerative and Neurometabolic Diseases, Department of Clinical Neurosciences, Fondazione IRCCS Istituto Neurologico Carlo Besta, Milan, Italy; 14 Neuromuscular Omnicentre (NEMO)-Fondazione Serena Onlus, Arenzano, GE Italy; 15 Department of Neuroscience, Reproductive and Odontostomatological Sciences, University of Naples “Federico II”, Napoli, Italy; 16 Unit of Neurology and Neuromuscular Diseases, Department of Clinical and Experimental Medicine, University of Messina, Messina, Italy; 17 Medical Genetics Unit, Azienda Ospedaliero Universitaria Di Ferrara, Ferrara, Italy; 18 Department of Neurosciences, University of Turin, Torino, Italy; 19 Division of Neurology and Neurorehabilitazion, IRCCS Istituto Auxologico Italiano, Piancavallo, VB Italy

**Keywords:** Attrv, Hereditary transthyretin amyloidosis, Pre-symptomatic genetic testing; PST

## Abstract

Hereditary transthyretin amyloidosis (ATTRv, v for variant) is a late-onset, autosomal dominant disease caused by progressive extracellular deposition of transthyretin amyloid fibrils, leading to organ damage and death. For other late-onset fatal diseases, as Huntington’s disease, protocols for pre-symptomatic genetic testing (PST) are available since decades. For ATTRv, limited experience has been reported to date, mostly gathered before the availability of approved therapies. We aimed at developing recommendations for a safe and feasible PST protocol in ATTRv in the era of emerging treatments, taking also into account Italian patients’ characteristics and healthcare system rules. After an initial survey on ongoing approaches to PST for ATTRv in Italy, two roundtable meetings were attended by 24 experts from 16 Italian centers involved in the diagnosis and care of this disease. Minimal requirements for PST offer and potential critical issues were highlighted. By November 2019, 457 families affected by ATTRv with 209 molecularly confirmed pre-symptomatic carriers were counted. The median age at PST was 41.3 years of age, regardless of the specific mutation. Half of the Italian centers had a multidisciplinary team, including a neurologist, an internist, a cardiologist, a medical geneticist and a psychologist, although in most cases not all the specialists were available in the same center. A variable number of visits was performed at each site. Experts agreed that PST should be offered only in the context of genetic counselling to at risk individuals aged 18 or older. Advertised commercial options for DNA testing should be avoided. The protocol should consist of several steps, including a preliminary clinical examination, a pre-test information session, an interval time, the genetic test and a post-test session with the disclosure of the test results, in the context of an experienced multidisciplinary team. Recommendations for best timing were also defined. Protocols for PST in the context of ATTRv can be refined to offer at risk individuals the best chance for early diagnosis and timely treatment start, while respecting autonomous decisions and promoting safe psychological adjustment to the genetic result.

## Background

Hereditary transthyretin amyloidosis (ATTRv) is an autosomal dominant, late-onset disease caused by the extracellular deposition of amyloid fibrils formed by misfolded transthyretin mutants [1, 2]. If untreated, the disease is fatal within 4–15 years from onset, according to genotype.

More than 130 different mutations of *TTR* have been identified worldwide [3]. The first-described Val30Met mutation [4] is the most frequent and may be associated with an early-onset phenotype, which is common in Portuguese population [5], or with a late-onset disease which has been described worldwide, including Italy [2, 6]. Moreover, few Italian regions (Sicily, Puglia, Lazio, Piedmont, Tuscan-Emilian Apennines) are endemic for specific mutations with variable age at onset and clinical presentation [7–10], mostly consisting of a mixed cardiologic and neurological phenotype. In most cases, the clinical onset is around the sixth decade of life [7–10].

For other late-onset neurodegenerative diseases, such as Huntington’s disease, familial frontotemporal dementia/amyotrophic lateral sclerosis and spinocerebellar ataxias, protocols for pre-symptomatic genetic testing (PST) are available since many years [11–14]. These counselling protocols govern the access to pre-symptomatic testing in order to protect participants against an unfavorable result, providing them with information about the disease and the psychosocial consequences of the test result. All these protocols aim to conjugate the respect for the autonomy with the maximum benefit, supporting the applicant in the decision making process of testing and helping her/him to cope with the results.

Based on this know-how and on their personal experience, a few years ago, experts of the European Network for TTR-FAP (familial amyloid polyneuropathy) proposed recommendations for pre-symptomatic genetic testing and managements of individuals at risk for ATTRv [15].

Even if most of those recommendations are still valid, during last years the ATTRv therapeutic scenario has rapidly changed with an increasing availability of drugs that target key molecular events of the amyloidogenesis process and are able to treat the disease [2]. Since all therapies are maximally effective in the early stages, the need to preserve at risk individuals from the psychological consequences of a positive test should be balanced against the possibility to offer them a close monitoring [16], prompting treatment start as soon as minor, but clinically meaningful disease signs, are detected.

However, it is difficult to predict treatment onset in mutation carriers and to define the optimal timing for pre-symptomatic testing. Genetic anticipation has been reported in early-onset Val30Met and significant variability in age of onset is observed also within families with non-Val30Met variants.

Recently, in order to determine when pre-symptomatic carriers of a TTR mutation should start a regular monitoring, it has been proposed to estimate the predicted age of onset of symptomatic disease (PADO). PADO depends on the specific TTR gene mutation, the typical age of onset for that mutation and the age of onset in other members of the proband's family. International expert consensus recommends that regular clinical monitoring should start 10 years before the PADO [16].

To promote equal, safe and effective access to PST for ATTRv in Italy, experts in the field gathered together to agree on best practices and optimal approach. In this paper, we address the medical, psychological, ethical, and legal issues related to PST for ATTRv and provide recommendations for optimal timing and setting to offer it.

## Methods

A preliminary questionnaire (available on request) was sent to all participants to evaluate the number of patients followed by each center, the number of at risk relatives identified, the number of pre-symptomatic carrier that developed ATTRv during follow-up monitoring, and the protocols locally in use for PST. Available literature on genetic counselling in ATTRv was also revised based on a PubMed search. We used the following search terms: “ATTR amyloidosis” together with “genetic counselling”; “pre-symptomatic”; “genetic tests” and “early diagnosis”.

In March and November 2019, two panel discussion meetings were attended by 24 ATTRv Italian experts to share experiences on PST practices, to identify the main controversial issues and to achieve a consensus on best PST procedures according to the Italian patients' population and healthcare system characteristics. These experts cover all Italian regions and include amyloid-specialized centers, genetics units as well as regional centers for rare neurological and cardiac diseases. In between and after the roundtables, experts circulated comments and contributions to achieve a shared draft. Finally, the consensus document, written by the scientific organizers on the basis of all individual inputs was sent to each expert for approval.

### Questionnaire results

By March 2019, the national Italian reference center in Pavia and the other 11 Italian centers followed 457 families affected by ATTRv. *TTR* mutations identified in the Italian population are very heterogeneous and include (according to historical nomenclature) Val30Met (112; 25%), Ile68Leu (104; 23%), Phe64Leu (68; 15%), Glu89Gln (56; 12%), Val122Ile (11; 2.5%), Tyr78Phe (2; 1%) and other variants in the remaining 21.5%. Pre-symptomatic carriers were 209, in most cases they had a parent (74%) and/or a sibling (33%) affected by ATTRv. PST was performed at a median age of 41 years and 4 months (range 30–57 years). In all centers the minimum age for testing was 18 years. During regular monitoring visits following PST, 26 pre-symptomatic carriers developed clinical manifestations of ATTRv amyloidosis. Mean latency between detection of the mutation and the onset of the disease was 51,08 months (median 51; standard deviation 38,52; range 6–144).

Half of the Italian centers had a multidisciplinary team in place but, in most cases, not all specialists were available at the same center. For instance, the geneticist was present in 7 centers, the psychologist in 6 centers, the cardiologist in 5 and an internist in 3 centers. In general, lack of a multidisciplinary team or of a particular specialist in a team, was due to the lack of resources, particularly in smaller centers, rather than the result of a specific choice. The structure of genetic counselling, i.e. the number of clinical evaluations including a pre-test visit, the test-visit and the delivery of the results, was variable within the centers according to different experiences and to the resources available.

All centers schedule annual follow-up visits for ATTRv carriers. These evaluations include neurological and cardiologic evaluations and usually begin approximately ten years before the PADO, in agreement with recent recommendations [16].

#### Ascertainment and basal clinical evaluation of at-risk subjects

When specialists diagnose a patient with ATTRv, they should also communicate that it is important to timely inform family members about the risk of disease recurrence. All relatives (siblings and offspring) should be considered as possible carriers of the mutated allele. ATTRv amyloidosis is a progressive and eventually fatal disease but treatment options are rapidly expanding and changing the natural history of the disease. Thus, the patient should be properly educated on the importance of informing at risk relatives about the disease, directing them to expert clinicians in ATTRv amyloidosis. Indeed, the patient and the relatives at risk must be aware that the earlier the treatment starts the better it is, as with current therapy it is difficult to substantially revert an already established damage.

The potential benefits of genetic testing are greater for siblings of an index patient than for the offspring. In fact, siblings of the index patients, especially those that are close to the predicted age of disease onset, are at higher risk for developing clinical disease in the immediate future and deserve the highest priority.

Occasionally genetic test performed in a sibling may reveal an ATTR cardiomyopathy instead of a previously misdiagnosed hypertensive or hypertrophic cardiomyopathy heart disease.

Adult subjects at risk of being carriers of *TTR* mutation should start monitoring not later than ten years before the PADO. Then, follow-up of genetically confirmed carriers will initially take place after 6 months, then every 12–24 months, and after it will become more frequent as the PADO approaches, especially for genotypes associated with more rapid disease progression (i.e. Val30Met, Gly47Glu, Ser77Tyr, Glu89Gln) [16].

Therefore, to appropriately deal with a PST request and to avoid possible diagnostic delays, it is relevant to first define whether a sibling is already symptomatic or not. A specific neurological and/or cardiologic evaluation can be offered also considering the phenotype of the mutation [2, 6]. Moreover, the clinician should explain overall features of ATTRv amyloidosis, the importance to undergo genetic analysis as well the significance of genetic results [6]. The following evaluations should be performed:

- *Neurological evaluation:* the at risk individual should be asked for possible diagnoses or clinical manifestations of carpal tunnel syndrome (CTS) [17] and lumbar spinal stenosis [18]. Symptoms and signs suggesting neuro-vegetative impairment should be recorded, such as pupillary abnormalities, dry eyes or dry mouth, abnormal sweating function, vasomotor signs (excessively cold hands and cold feet with discoloration), orthostatic intolerance (light-headedness, dizziness), gastrointestinal signs (nausea, vomiting, bloating, early satiety, abdominal colic, incontinence, or alternating diarrhea and obstinate constipation), genitourinary signs (urinary frequency and urgency, incontinence, or increased residual urinary retention), and sexual dysfunction (difficulty with erection and ejaculation) [19].
  Neurological examination should include mental state, cranial nerves assessments, examination of global and segmental strength, tone and bulk, sensory evaluation and deep tendon reflexes. Gait and balance should be also examined. Blood pressure should be measured in lying and standing position to detect orthostatic hypotension.
- *Cardiologic evaluation:* in ATTRv, symptomatic cardiac involvement is mainly characterized by heart failure (HF) with preserved ejection fraction [20]. The at risk individual should therefore be asked about symptoms and signs suggestive of HF, such as: dyspnea, fatigue, ankle swelling, dizziness [21]. Nevertheless, cardiac amyloidosis may result in a vast range of other manifestations, in particular atrial arrhythmias. It is therefore of primary importance that the evaluation of the at risk individual is performed by a cardiologist with expertise in ATTRv. A 12-lead ECG, a comprehensive echocardiographic evaluation according to the American Society of Echocardiography recommendations [22] and cardiac biomarkers measurements (natriuretic peptides and troponins) are the mainstay of cardiologic assessment and should always be performed. Such an evaluation represents the minimum core to be done in at risk siblings of an ATTRv subject.

After clinical and instrumental evaluations, the subject at risk is therefore defined as clinically affected or asymptomatic. If clinically affected, a diagnostic genetic test is firmly proposed. If it is asymptomatic, he/she can decide whether to access the protocol for PST.

### PST protocol: minimal steps and requirements

#### Information and consent

In the absence of an effective preventive therapy, individuals who undergo pre-test counselling should be ≥ 18 years old [23].

All people who may wish to take the test should be given updated and relevant information in order to make an informed voluntary decision. Pre-test counselling should include information not only on the test, but also about all the testing process.

Extreme care should be used when testing might provide information about a person, who has not requested the test (e.g. 25% at risk) [24]. Pre-symptomatic genetic testing is remarkable in that it is one of the few tests that can reveal important information about a third part who may not wish to have such information. This aspect should be drawn to the attention of the participant and discussed [11].

Informed consent for the test should be documented with the signature of the person to be tested and the professional responsible for the counselling as a standard medical practice.

The decision to take the test is the unique choice of the person concerned. No requests from third parts, be their family or otherwise, should be considered.

#### Laboratory and team requirements

Laboratories which perform genetic testing are expected to work under a quality assurance framework, meet rigorous standards of accuracy and participate in external quality assessment (EQA) schemes.

Nowadays there is increasing concern about the growing availability of commercial gene panels for human disease. It is fundamental to underline the need to follow established guidelines when performing genetic analysis. The entire process should involve teams with expertise in providing accurate interpretation of genetic data, patient counselling, and disease-specific management and follow up. This kind of support is not available in a commercial “genetic panel” that should therefore be avoided [25]. Consequently, PST for ATTRv must be proposed only within a solid framework of clinicians who guarantee multidisciplinary management, including the possible psychological impact of the test. Therefore, in addition to the referral clinician, a psychologist with experience in genetic counselling must be available from each team.

#### Test timing

Excluding exceptional circumstances, a minimum interval (e.g. one month) between the pre-test counselling session and the decision whether to take the test is recommended. Such an interval is necessary to give the person enough time to assimilate the pre-test information in order to make an informed and autonomous decision. The counsellor should ascertain that the pre-test information has been properly understood.

The patient should be aware of the waiting time for test, with a clear indication of the time between the sample being taken and disclosure of results. The entire process from pre-test counselling to disclosure of the results should not be longer than six months, unless the patient himself ask for more time [26].

The result of the predictive test should be delivered as soon as reasonably possible after completion of the test (preferably within one month from sample collection), on a date agreed upon in advance between the center, the counsellor, and the person. However, the person should have the option to ask for more time delay before receiving the results or also decide not to receive the results at all [23].

#### Disclosure and confidentiality of the results

The results of the test should be given personally by the counsellor to the person who requested the test. As a rule, members of the counselling team or the technical staff should not communicate any information concerning the test and its results to third parts without the explicit permission of the person tested (law 2019/2017).

If the genetic analysis is negative for mutation, clinicians must reiterate that the subject is healthy and accordingly she/he cannot transmit the disease to her/his offspring.

If the genetic analysis is positive, the clinicians must communicate and discuss the genetic result. At this stage, the need for psychological support should never be neglected.

#### Follow-up

During PST protocol and post-test contacts specific information on follow-up options should be provided, including (if applicable) participation in clinical research studies. The nature of emerging prodromal signs in mutation carriers and their management possibilities (if available) must be discussed.

If an adult asymptomatic at risk individual resulted positive at genetic test, we recommend a systematic follow-up which includes baseline visit and then, if symptoms suggestive of disease are not reported, we perform a first visit after 6 months and then, if patient is clinically unchanged, every 12–24 months, up to ten years before PADO. Then a regular visit every 6–12 months is requested.

At each visit, carriers should undergo blood tests including a full blood count, hemoglobin, renal function, albumin, troponin and NT-proBNP, and urine examination [15, 27]. Body weight should be measured at each examination, and BMI and mBMI calculated and monitored. For early detection of the neuropathy onset, a full neurologic evaluation is recommended. Clinical scales such as NIS (neuropathy impairment score), CADT (compound autonomic dysfunction test), Kumamoto scale, are useful tools for detecting early symptoms and signs. Nerve conduction studies to explore large fibers’ function and different tools to evaluate small nerve fibers may be employed. Autonomic function tests (such as tilt test, deep breathing, sweat tests), quantitative sensory tests, laser-evoked potentials, sympathetic skin responses or Electrochemical Skin Conductance (ESC) and skin biopsy are all effective tools for the early detection of small fiber neuropathy [16, 28, 29]. Cardiac evaluation should include ECG, 24-h Holter monitoring and echocardiographic study. In the case of otherwise unexplained abnormalities in the ECG, echocardiogram study and/or biomarkers, even in the absence of symptoms suggestive of cardiac ATTRv, it should be recommended to reach a definitive diagnosis [15, 16]. Scintigraphy with bone tracers 99mTc-labeled 3,3-diphosphono-1,2 (DPD) propanodicarboxylic acid, 99mTc-labeled pyrophosphate (PYP) or 99mTc-labeled pyrophosphate (PYP) [30, 31] but not 99mTc-labeled methylene diphosphonate (99mTc-MDP]MPD) [32, 33] allows diagnosis of transthyretin-related cardiac amyloidosis, in many cases even without the need of biopsy [31]. However, it is necessary to report the low sensitivity of bone scintigraphy in some TTR mutations such as Phe64Leu in which, even in the presence of clear clinical, biochemical and echocardiographic signs of cardiac involvement, cardiac uptake can be absent [34]. In these cases, the use of cardiac magnetic resonance may represent a valid alternative [35]. Notably, bone scintigraphy may identify amyloid deposition before the development of abnormalities on echocardiography [36]. However, performing bone scintigraphy without other suggestive symptoms or signs of ATTRv is not recommended, since it is not included in the guidelines for the diagnosis of ATTRv and this is not routinely carried out in our centers.

Finally, a full ophthalmologic examination should be performed for the early detection of eye involvement (vitreous opacities, glaucoma) [37] and other specialists (i.e. gastroenterologist or nephrologist) consultation may be required if specific symptoms are reported by the carriers [2, 6].

## Final remarks

- A referral physician who will take care of the pre-symptomatic carrier management should be identified in any referral center. The referral professional should discuss and provide the pre-symptomatic carrier with a roadmap of management and follow-up targeted to the detection of early symptoms and/or signs that indicate the initial clinical manifestation of ATTR amyloidosis, so to start early a proper therapy.
  Although the results of genetic tests should be better discussed in a team context, this is unfeasible in the majority of settings. Therefore, depending on the center, the referral physician may be a neurologist, a cardiologist, a medical geneticist, as long as she/he has an expertise in genetics, clinical features and management of ATTRv.
- A multidisciplinary collaboration is always warranted, with a defined path of care. According to expert consensus the key clinical parameters should be established at the baseline, then the timing and frequency of the clinical and laboratory follow-up should be tailored according to symptoms and signs associated with any specific mutation. The follow-up of pre-symptomatic carrier should include a multidisciplinary assessment comprehensive of neurological, cardiologic, ophthalmologist, gastrointestinal and, on demand, also nephrological evaluation.
- Pre-symptomatic carriers of *TTR* mutation should start regular monitoring about ten years before PADO. Pre-symptomatic carriers should also be instructed on the symptoms of the disease, to be alerted and ready to contact the treating physician also outside of the scheduled visits, if needed. Early recognition of symptoms/signs of neuropathy (large and small fibers) is crucial since the indication to start a specific treatment (both with stabilizers or new generation therapies) is almost entirely dependent on the demonstration of the neuropathy.
- Newly identified pre-symptomatic carriers should be taken in charge and followed up regularly after the return of the diagnostic test, both to monitor their reaction to the communication of the diagnosis, and to detect the first signs and symptoms of the disease, thus allowing timely therapy starting.
- If, in one of the test steps or subsequently the need for specific psychological support is identified, access to a psychologist with specific expertise in PST must be guaranteed. It is important to allow it also to the subjects for whom the mutation has been excluded with at least one follow up visit as they might develop a negative reaction (survivor guilt) [38].

All final remarks are summarized, as brief practical recommendations, in Table 1.

**Table 1:** Practical recommendations

Identification of only one clinical referent in each center	
Necessity of comprehensive multidisciplinary assessment	
Regular monitoring of pre-symptomatic ten years before PADO	
Taking in charge newly identified pre-symptomatic carriers	
Specific psychological support after pre-symptomatic testing in selected cases	

## Data Availability

All the data generated during the current study are available from the corresponding author on reasonable request.

## Abbreviations

ATTRv: Hereditary transthyretin amyloidosis, v for variant
CADT: Compound autonomic dysfunction test
CTS: Carpal tunnel syndrome
EQA: External quality assessment
ESC: Electrochemical skin conductance
FAP: Familial amyloid polyneuropathy
HF: Heart failure
NIS: Neuropathy impairment score
PADO: Predicted age of onset of symptomatic disease
PST: Pre-symptomatic genetic testing
